# Predictive Modeling of Mechanical Properties of Silica Fume-Based Green Concrete Using Artificial Intelligence Approaches: MLPNN, ANFIS, and GEP

**DOI:** 10.3390/ma14247531

**Published:** 2021-12-08

**Authors:** Afnan Nafees, Muhammad Faisal Javed, Sherbaz Khan, Kashif Nazir, Furqan Farooq, Fahid Aslam, Muhammad Ali Musarat, Nikolai Ivanovich Vatin

**Affiliations:** 1 Department of Civil Engineering, Abbottabad Campus, COMSATS University Islamabad, Abbottabad 22060, Pakistan; afnan@cuiatd.edu.pk (A.N.); isherbazkhan@gmail.com (S.K.); furqan@cuiatd.edu.pk (F.F.); 2 Department of Civil Engineering, School of Engineering, Nazabayev University, Astana 010000, Kazakhstan; kashif.nazir@nu.edu.kz; 3 Military Engineering Service (MES), Ministry of Defence (MoD), Rawalpindi 43600, Pakistan; 4 Faculty of Civil Engineering, Cracow University of Technology, 24 Warszawska Str., 31-155 Cracow, Poland; 5 Department of Civil Engineering, College of Engineering in Al-Kharj, Prince Sattam Bin Abdulaziz University, Al-Kharj 11942, Saudi Arabia; 6 Department of Civil and Environmental Engineering, Bandar Seri Iskandar 32610, Perak, Malaysia; muhammad_19000316@utp.edu.my; 7 Peter the Great St. Petersburg Polytechnic University, 195251 St. Petersburg, Russia; vatin@mail.ru

**Keywords:** green concrete, industrial waste, predictive modeling, machine learning, cross-validation, sensitivity analysis

## Abstract

Silica fume (SF) is a mineral additive that is widely used in the construction industry when producing sustainable concrete. The integration of SF in concrete as a partial replacement for cement has several evident benefits, including reduced CO_2_ emissions, cost-effective concrete, increased durability, and mechanical qualities. As environmental issues continue to grow, the development of predictive machine learning models is critical. Thus, this study aims to create modelling tools for estimating the compressive and cracking tensile strengths of silica fume concrete. Multilayer perceptron neural networks (MLPNN), adaptive neural fuzzy detection systems (ANFIS), and genetic programming are all used (GEP). From accessible literature data, a broad and accurate database of 283 compressive strengths and 149 split tensile strengths was created. The six most significant input parameters were cement, fine aggregate, coarse aggregate, water, superplasticizer, and silica fume. Different statistical measures were used to evaluate models, including mean absolute error, root mean square error, root mean squared log error and the coefficient of determination. Both machine learning models, MLPNN and ANFIS, produced acceptable results with high prediction accuracy. Statistical analysis revealed that the ANFIS model outperformed the MLPNN model in terms of compressive and tensile strength prediction. The GEP models outperformed all other models. The predicted values for compressive strength and splitting tensile strength for GEP models were consistent with experimental values, with an R^2^ value of 0.97 for compressive strength and 0.93 for splitting tensile strength. Furthermore, sensitivity tests revealed that cement and water are the determining parameters in the growth of compressive strength but have the least effect on splitting tensile strength. Cross-validation was used to avoid overfitting and to confirm the output of the generalized modelling technique. GEP develops an empirical expression for each outcome to forecast future databases’ features to promote the usage of green concrete.

## 1. Introduction

Global warming is widely believed to be the primary contributor to greenhouse gas (GHG) emissions, with CO_2_ being the most abundant and strongest influence of all GHGs [1,2]. Around 5–7 percent of global CO_2_ emissions are attributed to the cement industry [3]. Due to its mechanical and durability features, concrete is a widely used building material [4]. Around 8% of CO_2_ emitted during the concrete making process contributes to global warming [4,5]. Concrete is expected to be manufactured at a rate of 20 billion tons per year, making it the second most frequently utilized substance on the planet after fresh water. Apart from its benefits, concrete is harmful to the Earth and human health and has long-term negative consequences on the natural environment and atmosphere [6]. It expands the human footprint by creating living space out of thin air, spreading across fertile topsoil, and impeding biodiversity. The biodiversity crisis is the primary focus of research, as it is one of the most serious dangers to a sustainable ecosystem and is mostly caused by urbanization. For hundreds of years, humans have longed for the dubious benefits of concrete to overlook this environmental drawback. However, the balance is currently shifting in the opposite direction. At times of unsettling transition, solidity is an alluring quality that can create more problems than it can resolve [7].

The most energy-intensive stage of the cement manufacturing process is clinker production, which accounts for half of all CO_2_ emissions from concrete (produced by calcareous and clay minerals in the kiln). Nearly 900 kg of CO_2_ is emitted during the manufacturing of a ton of cement. It must be heated to extremely high temperatures during the cement manufacturing process to generate clinkers. Cement is made by grinding clinker to a fine powder and then mixing it with gypsum (Ca_3_SiO_5_), sometimes called alite. It is generated during the clinker production process and gives an excessive early strength. Alite must be maintained at a temperature of 1500 °C during this process [8,9]. According to some studies, alite can be replaced by various naturally occurring minerals that require a lower roasting temperature than alite. Carbon emissions from concrete have long been a source of concern for both the academic and industrial sectors [10]. Numerous techniques have been proposed to address this issue, one of which asserts that we can achieve sustainability by completely or partially replacing cement with readily available natural materials [11,12,13]. Extra cementitious materials such as silica fume (SF) have been employed to partially replace cement in concrete mixtures to offset the cement industry’s CO2 emissions [14,15,16,17]. SF is a significant by-product of the silicon metal manufacturing sector. Silicon metal is a semi-metallic element that exhibits various metal-like properties. After oxygen, silica is the second most abundant element in the Earth’s crust, occurring primarily in the form of silicon dioxide or silicates but also in its pure state [18].

**Figure 1:**
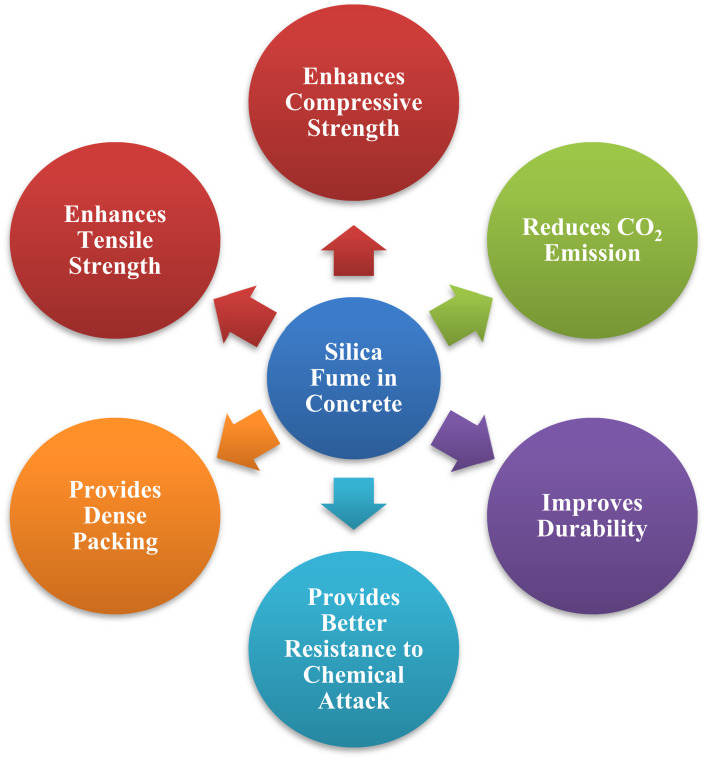
Silica fume benefits in concrete.

SF is a toxic substance that has a deleterious effect on the atmosphere and its surroundings. Until the mid-1970s, nearly all silica fume was released into the environment. As environmental worries about SF grew, it was being used in a variety of applications. SF is composed of extremely small particles and a high concentration of amorphous silicon dioxide, making it a highly pozzolanic material. Due to its amorphous structure, it is extremely reactive. They are spherical and possess a substantial surface area. Because SF particles are 100 times smaller than cement particles, they are entirely packed with cement grains and also react with calcium hydroxide to generate more CSH, resulting in the earlier strength [16,19,20,21]. Due to its small size, it enhances the packing density of concrete. They are also significantly more advantageous in terms of strength. Additionally, due to its superior qualities, SF concrete has been widely employed in high-strength and high-performance concrete for highway bridges, maritime structures, and parking decks [14,22,23]. Figure 1 represents the advantages of silica fume in concrete.

Numerous experimental studies have been conducted to determine concrete’s short- and long-term mechanical characteristics when various quantities of fine aggregate or cement are replaced with SF [24,25]. According to the literature, replacing 15% of the SF content improves the mechanical properties of SF, including compressive strength, initial strain owing to creep, and modulus of elasticity. However, higher concentrations result in a reduction in concrete creep over time [26]. The strength development of SF-based concrete is temperature-dependent, material size-dependent, and silica content-dependent. Between 3 and 28 days is when the majority of the strength contribution occurs at a typical curing temperature. After 28 days, the additional strength conferred by SF is negligible. The substitution of SF for cement in the range of 5–25% with a water to binder ratio of 0.26–0.42 increases compressive strength by approximately 6–30% [13]. The compressive strength of silica fume concrete (SFC) can be greatly enhanced by adjusting the water–cement ratio between 10% and 20% [26]. Increases in the water–cement ratio of SFC resulted in a decrease in the overall strength of concrete. The compressive strength of concrete is reduced by 27% after 28 days when the water–cement ratio increases by 0.05% with a 15% SF concentration [27]. Numerous factors affect the qualities of concrete, including the proportions of cement, sand, aggregate, and water. The proportion of these components in concrete impacts its strength and durability. The mechanical characteristics of concrete exhibit anomalous behavior at various mix ratios incorporating additives including silica fume. To accommodate this behavior and to facilitate the widespread use of SF in concrete, a link between the mechanical properties of SF and the proportion of materials used in concrete is required to support sustainable development. To develop this link, various artificial intelligence modelling approaches are used, and empirical models are constructed to promote sustainable growth. SFC design must take into account fundamental mechanical qualities such as compressive strength and splitting tensile strength.

Additionally, SFC mixtures must be cost-optimized to attain desired attributes by the effective proportioning of SFC components. Traditionally, laboratory-prepared test batches have been used to ensure that these criteria are met, and that building specifications are followed [28]. Because a laboratory can only produce a finite number of tests, experimental procedures can generate well-performing, rather than best-performing, quantities of SFC combinations. Computational modelling approaches may be a viable alternative to laboratory-based mixture optimization due to their time-saving nature. These approaches begin by developing objective functions between the inputs (concrete ingredients) and outputs (properties), and then employ optimization algorithms to determine the optimal concrete mixes. Traditionally, goal functions have been developed for linear or nonlinear models [29]. However, because the relationships between concrete properties and controlling factors are highly nonlinear, the coefficients of these models cannot be determined exactly [5]. As a result, researchers are employing machine learning (ML) approaches to predict concrete qualities.

Historically, various machine learning techniques have been used to predict concrete qualities such as the modulus of elasticity, compressive strength, splitting tensile strength, and so on. Among machine learning algorithms, multilayer perceptron neural networks (MLPNNs) [30,31,32,33,34], support vector machines (SVMs) [35], genetic engineering programming (GEPs) [36,37,38,39,40], and deep learning (DL) [41,42,43] were the most often utilized. Ling et al. [44] used SVM in conjunction with k-Fold cross-validation, an artificial neural network (ANN), and a decision tree (DT) to forecast concrete strength degradation in a marine environment. SVM anticipated the expected results with greater accuracy and demonstrated superior performance compared to the other two approaches. Additionally, Motamedi et al. [45] extended the SVM-based analysis to a more sophisticated screen and determined the unrestricted compression capacities of cement-sand cockle-coated combinations. Chithra et al. [46] developed an ANN technique for predicting the strength of copper slag and nano-silica concrete. Tanyildizi et al. [47] used SVM and ANN to predict lightweight concrete’s compressive and flexural strengths reinforced with carbon fiber. The ANN approach achieves a higher level of accuracy, with R^2^ values of 0.99 and 0.96 for compressive and flexural strength, respectively. Naderpour et al. [48] used ANN to forecast the compressive strength of recycled aggregate concrete and building waste concrete.

Similarly, Kadir et al. [49] predicted compressive strength using ANN, DT, SVM, and linear regression approaches. It was discovered that the DT approach accurately predicted compressive strength values and outperformed others. Awoyera et al. [50] used GEP and ANN to construct models to forecast the strength properties of geopolymer self-compacting concrete made from raw ingredients. The author found that the GEP model outperformed the ANN model by providing an empirical relationship for predicting output parameters. Similarly, Ziolkowski et al. [51] investigated the role of ANNs in forecasting concrete’s compressive strength. Mathematical equations for creating the aforementioned output were generated. Chopra et al. [52] used DT, RF, and ANN to forecast the compressive strength of concrete for 28, 51, and 90 days. Statistical parameters including the coefficient of determination (R^2^) and root mean square error (RMSE) were used for the evaluation of models. Based on these statistical indicators, it was concluded that RF predicted the best results, followed by ANN. Han et al. [53] also highlighted the utility of machine learning algorithms for calculating the strength of reinforced concrete materials with high accuracy. Similarly, Table 1 outlines research on machine learning conducted by researchers employing waste materials.

The novelty of this study is two-fold. First of all, the compressive and tensile strengths of SFC were predicted using MLPNN and ANFIS. Secondly, a GEP model was constructed for each outcome, comprising equations and an expression tree. Then, machine learning techniques were compared to the GEP model. According to the authors, there is no comparable study in the literature that uses ensemble machine learning and GEP for SFC. Numerous statistical variables were employed to evaluate the predictive accuracy of machine learning algorithms. This project aims to promote the use of SF in concrete and to perform investigations focusing on carbon footprint reduction. We want to make concrete more environmentally friendly by employing computational tools to optimize the use of SF as an additive or a replacement material in concrete for more sustainable development. This article discusses the use of advanced machine learning algorithms to investigate the behavior of SFC and how they were used to develop the most environmentally friendly concrete.

## 2. Algorithms for Machine Learning

**Figure 2:**
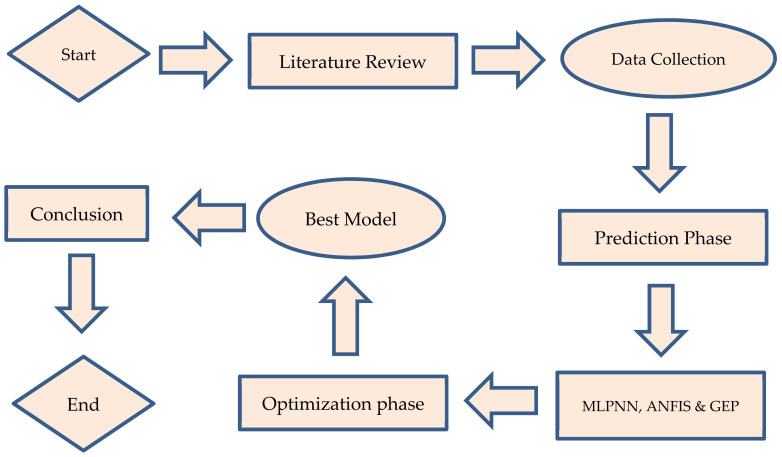
Machine learning algorithms.

Machine learning (ML) is a relatively new topic of artificial intelligence that is commonly utilized in the construction industry to forecast the behavior of materials [5]. As indicated in Figure 2, the current work applied machine learning techniques to estimate the compressive and split tensile strengths of SFC by applying MLPNN, ANFIS, and GEP. These approaches are deemed to be the most effective data prediction algorithms and were chosen based on their widespread use in similar research. The next section provides an overview of the AI and machine learning methodologies used in this research.

Machine learning models are extremely efficient in terms of computation and processing time. When compared to traditional models, it reduces error rates to virtually insignificant levels. The research establishes an empirical model between the mechanical properties of SFC and the mix proportions using various machine learning approaches and then compares the findings to forecast the best model among them. Among the major machine learning techniques, this article discusses MLPNN (a kind of ANN), ANFIS, and GEP. The following section briefly discusses the various modelling strategies employed in this study.

### 2.1. Multilayer Perceptron Neural Network (MLPNN)

The MLPNN algorithm consists of neurons, which are called the perceptron. These networks are based on a single output from multiple inputs by producing nonlinear mapping among input and output vectors. The complex neural networks are based on the basic multilayer perceptron (MLP) model. The capacity to predict neural networks derives from the hierarchical or multi-layered network structure [70].

**Figure 3:**
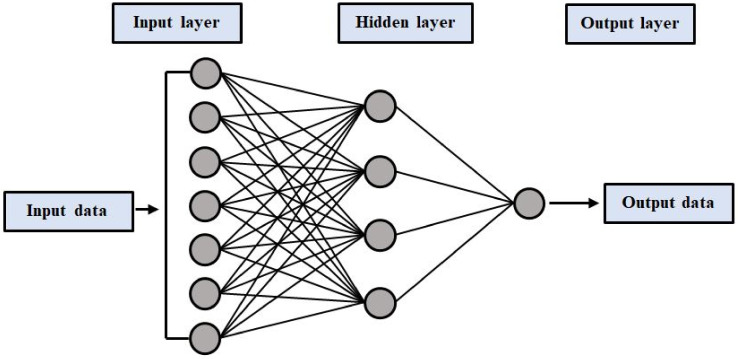
Typical neural network architecture.

These networks consist of three steps; in the forward pass, the model input is forwarded and multiplied by weight, the bias is applied to each sheet, and the measured model output is determined. Predicted outputs are evaluated that match the given inputs. Loss is determined after the forward exit. The output model provides predicted results after taking the input parameters. The failure can be compared with predicted results by means of the back-propagation algorithm. Different loss functions are used depending on our performance and requirements. The backward pass propagates partial derivatives of the cost function concerning the various parameters back into the network. In this process, there is back-propagation loss and the weights of the model are updated using gradient descent. An MLP contains a minimum of three layers of nodes: an input, hidden, and output layer. This network has three layers: the input layer on the left side with three neurons, the hidden layer in the middle with three neurons with nonlinear mapping, and the output layer on the right with one neuron, as seen in Figure 3.

### 2.2. Adaptive Neural Fuzzy Detection System (ANFIS)

Jang was the first to introduce the adaptive network-based fuzzy inference technology. It is a remarkable computational intelligence model that combines the learning capabilities of ANNs with the reasoning capabilities of fuzzy logic. ANFIS has a better estimating ability and is a better approach for computing nonlinear complicated problems with greater precision. ANFIS uses input–output sets and a series of IF–THEN fuzzy rules to incorporate the human-like reasoning style of fuzzy inference systems (FIS). FIS contains structured knowledge in which each fuzzy rule defines the system’s local behavior, but it lacks the adaptability to deal with a changing external environment. As a result, FIS has been updated to include neural network learning techniques, resulting in ANFIS. The basic learning method of the network, back propagation, tries to reduce the prediction error. The learning skills of a neural network and the reasoning capabilities of fuzzy logic were integrated in ANFIS for the reasons stated above. The ANFIS model is especially effective in a variety of engineering applications when data is inconsistent or nonlinear, where conventional methods fail or are too complicated to employ. Various adjustments were done in order to produce the most efficient ANFIS model with the minimum possible error size.

**Figure 4:**
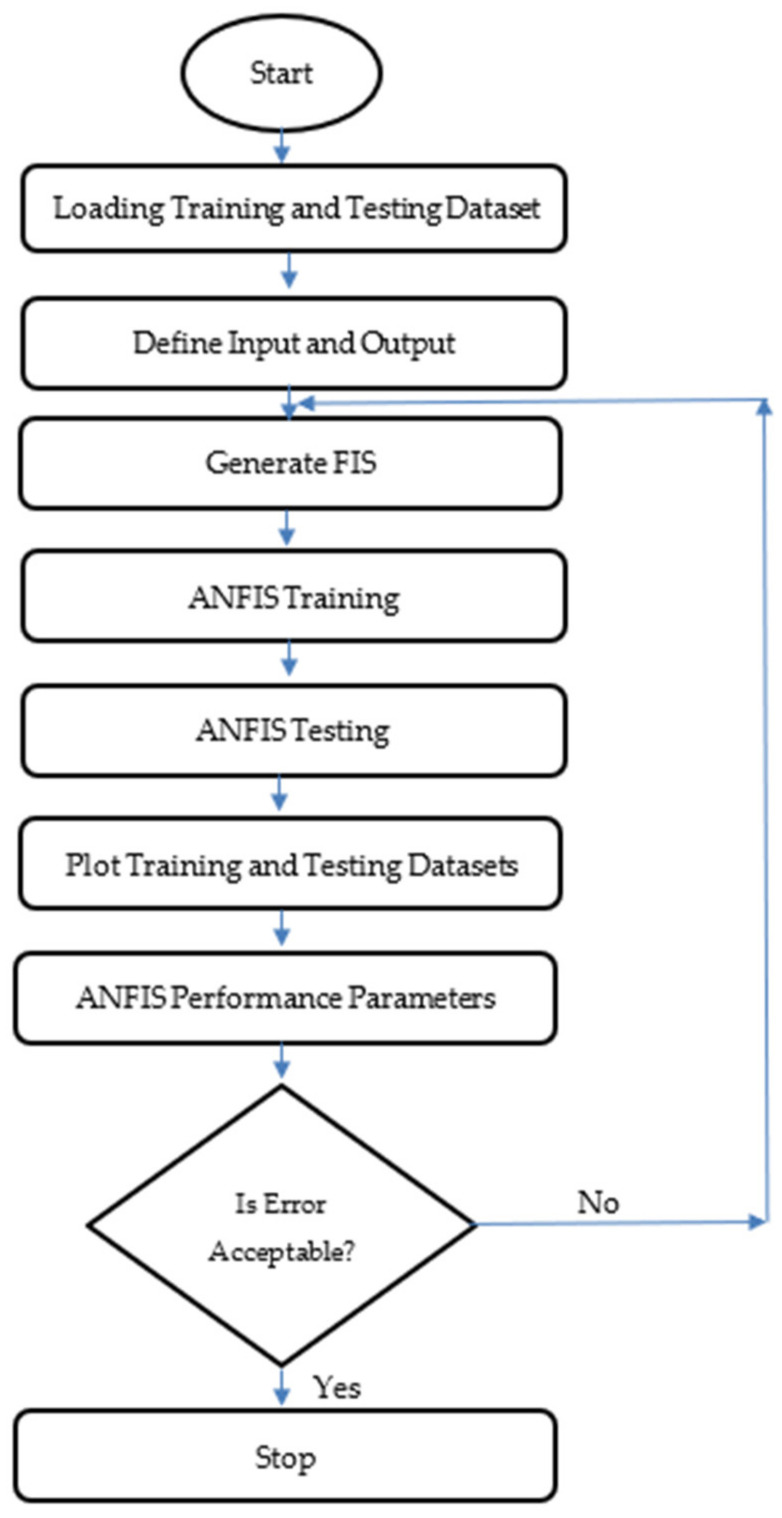
Flow chart of ANFIS.

ANFIS is made up of various layers, as shown in Figure 4. ANFIS structure with two inputs and one output is shown in this diagram, which is made up of four membership functions and four rules. According to the ANFIS structure shown in Figure 4, the layer structure of ANFIS is detailed below. The first layer is known as the fuzzification layer. To generate fuzzy clusters from input values, the fuzzification layer employs membership functions. The rule layer is the second layer. The rules’ firing strengths are calculated using membership values derived in the fuzzification layer. The normalizing layer is the third layer. It determines the normalized firing strengths of each rule. The ratio of the i^th^ rule’s firing strength to the total of all firing strengths is the normalized value. The fourth layer is known as the defuzzification layer. In each node of this layer, weighted values of rules are determined. The summation layer is the fifth layer. The actual output of ANFIS is obtained by summing the outputs obtained for each rule in the defuzzification layer.

### 2.3. Genetic Algorithm and Gene Expression Programming

**Figure 5:**
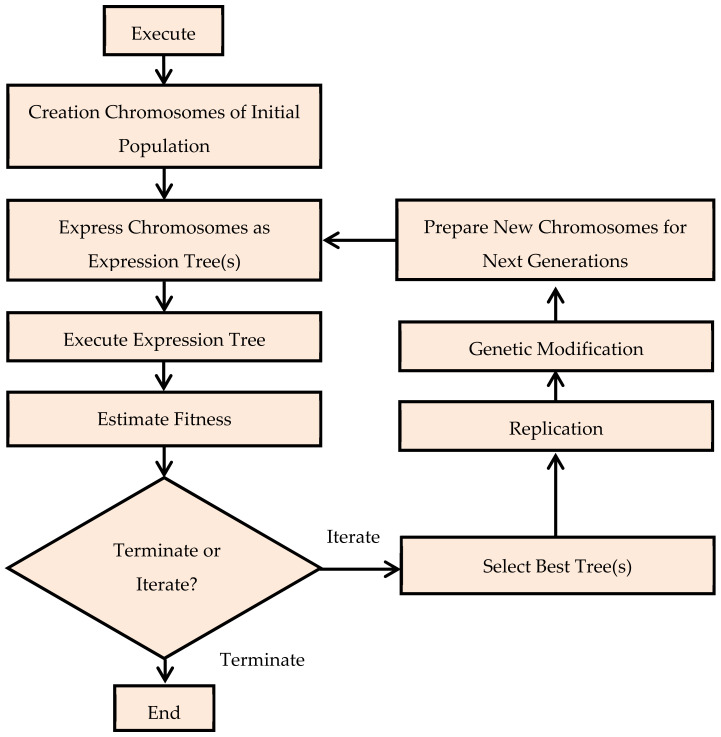
Flow chart of the GEP [73].

Genetic algorithms (GA) were first introduced by John et al. and are known as genetic expression [71]. The introduction of GA in the field of artificial intelligence is evolutionary and has overcome many limitations. GA are computer-based models that work on the evolution mechanism of nature. GEP consists of two primary parts known as tree expression (ET) and chromosomes [71]. The mathematical information or function is encoded in the chromosome and then this information is used to build the initial chromosome which is then converted into ETs [72]. These expression tree values are translated by means of language used and written in the form of mathematical expression [73]. The GA starts with the random creation of the chromosome based on individual programs [74]. Subsequently, the fitness of each chromosome is evaluated; based on fitness results, the procedure of reproduction is applied until the best chromosome is obtained. This chromosome is then extracted to pass them to the next generation. This process is continued unless the chromosome with the best survival and the best fitness value is obtained. The GEP model contains five parameters having the same analogy to GP, including terminal set, fitness function, control parameters, function set, and terminal conditions. The basic steps and parameters are represented in Figure 5**.**

## 3. Modeling Dataset and Model Development

**Figure 6:**
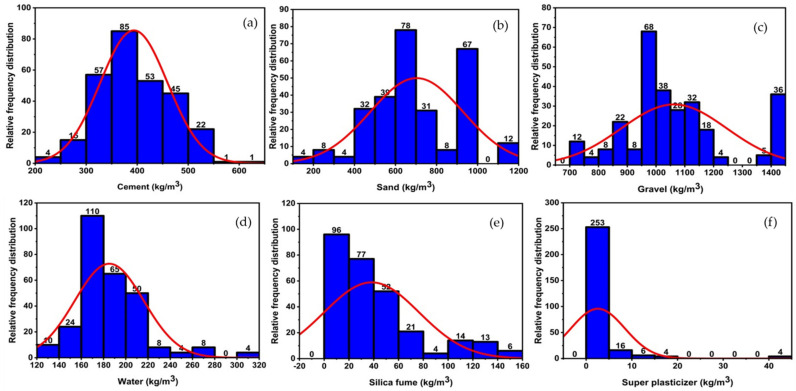
Relative frequency distribution of parameters to compressive strength; (**a**) cement, (**b**) sand, (**c**) gravel, (**d**) water, (**e**) silica fume, (**f**) super plasticizer.

**Figure 7:**
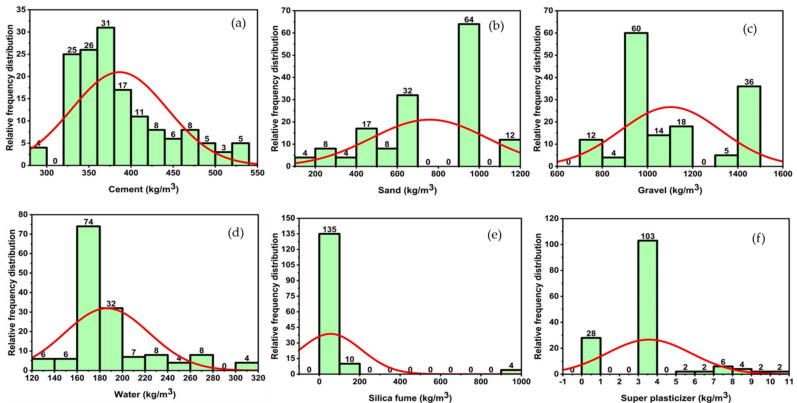
Relative frequency distribution of parameters to tensile strength; (**a**) cement, (**b**) sand, (**c**) gravel, (**d**) water, (**e**) silica fume, (**f**) super plasticizer.

The silica fume concrete (SFC) database was constructed using data from 22 internationally published investigations [26,28,75,76,77,78,79,80,81,82,83,84,85,86,87,88]. Figure 6 and Figure 7 illustrate the frequency distribution and statistical description of the database, which contains 283 compressive tests (fc) and 149 split tensile strength tests (fst). The mean, standard deviation, median skewness, maximum and minimum ranges of metrics, as well as the maximum and minimum ranges of parameters, are reported in Table 2 and Table 3. Gandomi et al. [89] recommended that the minimal ratio between the input variables and the database be three, and that it should be greater than five for reliable models. The ratios are substantially higher in this work using 283 databases for compressive strength and 149 databases for split tensile strength with six input factors, i.e., 47.17 and 24.83, respectively. Prior to constructing a model, the primary process that influences the SFC’s attributes is input selection. The constituents with the greatest influence on the properties of concrete are isolated in order to construct a generalized function. Concrete properties are studied as a function of Equation (1).

(1) $$\mathrm{fc},\mathrm{fst}\;\left(\mathrm{MPa}\right)\;f\left(C,\mathrm{FA},\mathrm{CA},W,\mathrm{SF},\mathrm{SP}\right)$$

These variables have an effect on the model’s strength forecast. With the intended output (f’c and fst), the relationship between these input variables is calculated. Table 4 and Table 5 list the lowest and maximum ranges of input variables that are functions of outputs, along with their ranges. Other variables also affect the characteristics of concrete, although their effect on the desired output of SFC is minor. Machine-learning empirical models were developed using training data (80% of total data) and then applied to validation data (20% of total data) to determine the model’s precision and accuracy [90]. The database, which was compiled from the literature, provides data on the % of SF replacement, water-to-binder ratios, the specific gravity of fine aggregate and SF, the fineness modulus of SF, and the superplasticizer fractions used to preserve workability. A database’s training set is utilized to build a model, whereas the built-in model is evaluated using test data or a validation set [5].

## 4. Models Evaluation Criteria

The performance of the developed model on a training or testing set can be quantified using statistical errors such as mean absolute error (MAE), root mean square error (RMSE), root mean squared logarithmic error (RMSLE), and root square value (R^2^). However, the R^2^ value, also known as the coefficient of determination, is considered the best of these for model evaluation. With advancements in the field of artificial intelligence, several modelling techniques have been used to construct prediction models for the resulting concrete’s mechanical properties. To promote the use of SF in concrete on an industrial scale, we attempted to develop regression and GEP models between compressive strength and split tensile strength with a mixed proportion of SFC and then compared them to determine the model that best predicts the output with the least or no deviation. The models are tested in this work using statistical analysis and the computation of error metrics. These metrics can provide a variety of insights regarding the flaws in your model. Additionally, the coefficient of variance and standard deviations are used to evaluate the model’s performance. The correctness and validation of the model are justified in this study by its coefficient of determination. R^2^ values between 0.65 and 0.75 indicate satisfactory findings, whereas values less than 0.50 indicate disappointing results. R^2^ can be determined using Equation (2).

(2) $$R^{2}=\frac{\sum_{i=1}^{n}\left(M_{i}-{\bar{M}}_{i}\right)(P_{i}-{\bar{P}}_{i})}{\sqrt{\sum_{i=1}^{n}{\left(M_{i}-{\bar{M}}_{i}\right)}^{2}\sum_{i=1}^{n}{\left(P_{i}-{\bar{P}}_{i}\right)}^{2}}}$$

MAE is the mean of absolute error when all input entities have the same weight; it is the difference between prediction and observed value. To remove the negative sign, the absolute value is used. It calculates the absolute size of the errors and uses the same units as the result. A model with an MAE value inside a range can have extremely large errors at times. It is determined using Equation (3).

(3) $$\mathrm{MAE}=\frac{1}{n}\sum_{i=1}^{n}\left|P_{i}-M_{i}\right|$$

The RMSLE algorithm takes into account the relative inaccuracy between the anticipated and actual values. It is defined as the discrepancy between the expected and actual logarithms of a value. RMSLE is calculated using Equation (4), where x is the predicted value and y denotes the actual value. This is advantageous when dealing with right-skewed outputs, as the log transform restores the target spread to its original state.

(4) $$\mathrm{RMSLE}=\sqrt{\frac{1}{N}\overset{N}{\underset{i=1}{\sum }}{\left(\log\left(\mathrm{yi}+1\right)-\log\left(\hat{y}+1\right)\right)}^{2}}$$

The root mean square error (RMSE) is the average of the squared differences between estimation and actual measurement. It quantifies the error’s mean square magnitude. It is the expected error’s standard deviation. Large exceptions, such as outliers, are given greater weight in this approach, resulting in larger squared differences and smaller squared differences. The root mean square error quantifies the model’s average prediction error while predicting the output for a given input. The lower the root mean square error, the more accurate the model. The RMSE score of 0.5 indicates that the model is unable to reliably predict the data. The root mean square error (RMSE) can be calculated using Equation (5). Table 6 summarizes the various statistical parameters.

(5) $$\mathrm{RMSE}=\sqrt{\frac{\sum_{i=1}^{n}{\left(P_{i}-M_{i}\right)}^{2}}{N}}$$

## 5. Results and Discussion

### 5.1. Formulation of the Compressive Strength and Split Tensile Strength of SFC

By integrating and combining several inferior analytical models, machine learning techniques tend to alleviate excessive training concerns (sub-models). By carefully altering training data, the development of multiple sub-models/classification components (1, 2,…, m) will aid in the development of a more proficient learner. More precisely, the optimal parametric/predictive model can be created by combining qualifying sub-models with averaging/voting procedures. Compressive and split tensile strength models were built and assessed in this study using determination coefficient (R^2^) values. R^2^ values for several models are shown in Table 7 for each outcome.

#### 5.1.1. Model Outcome of MLPNN

**Figure 8:**
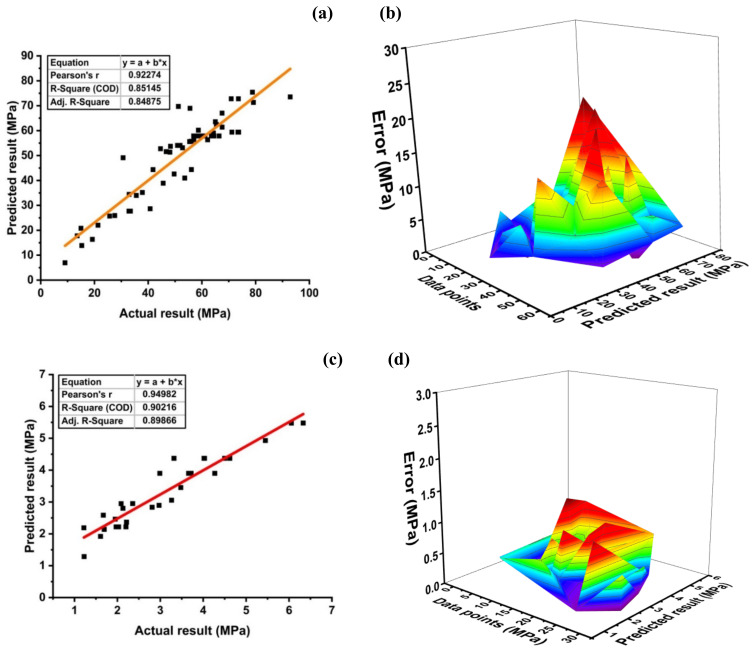
MLPNN model for; (**a**) compressive strength and (**b**) its error distribution between; (**c**) splitting tensile strength and (**d**) its error distribution.

Figure 8a depicts the compressive strength prediction of the compressive strength MLPNN model of SFC with R^2^ = 0.85. The error distribution of the targeted values with the model values is shown in Figure 8b. The MLPNN compressive model shows an average error of 5.28 MPa, with the maximum and minimum errors of 19.34 MPa and 0.048 MPa. Moreover, data indicate imprecision of 57.90% below 5 MPa, 24.56% between 5–10 MPa, 12.28 percent between 10–15 MPa, and 5.26 % between 15–20 MPa. 

The MLPNN split tensile strength model depicts R^2^ = 0.90 as shown in Figure 8c. The error distribution of the models obtained is depicted in Figure 8d. The MLPNN tensile strength model shows an average error of 0.41 MPa with maximum and minimum error values of 1.05 MPa and 0.02 MPa, respectively. In addition, 96.67% of error is below 1 MPa, and only 3.34% of the error lies in between 1 to 2 MPa. The results of the model are satisfactory and can be used to predict the split tensile strength of the model. Based on the above stats, the MLPNN is capable of predicting the compressive strength and split tensile strength of SFC with appreciable accuracy.

#### 5.1.2. Model Outcomes of ANFIS

**Figure 9:**
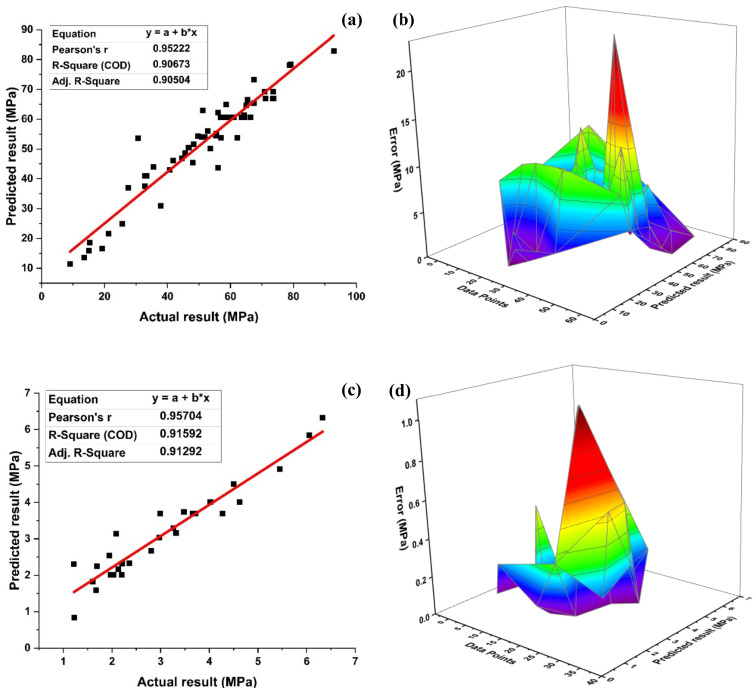
ANFIS model for (**a**) compressive strength and (**b**) its error distribution between actual and target values; (**c**) split tensile strength model and (**d**) its error distribution between actual and target values.

Figure 9a shows the relation of predicted values with the target values with R^2^ = 0.91 for ANFIS compressive strength. Figure 9b depicts maximum and minimum errors of 22.94 MPa and 0.001 MPa, respectively, with an average error of 4.18 MPa for the ANFIS compressive strength model. Moreover, data indicates an error of 71.93% below 5 MPa, 21.05% between 5–10 MPa, 5.26% between 10–15 MPa, 0% between 15–20 MPa and 1.75 % between 20–25 MPa. The error distribution of MLPNN shows a higher peak in comparison to the ANFIS model but the overall performance of both models gives approximately the same results.

Figure 9c shows R^2^ = 0.92 for the ANFIS tensile strength model. The average, maximum and minimum errors observed from Figure 9d are 0.26, 1.09, and 0.001 MPa, respectively, for the ANFIS split tensile strength model. Data of the ANFIS tensile strength model shows an error of 93.33% below 1 MPa, whereas a 6.67% error between 1–2 MPa. The ANFIS model can predict the targeted results with an ignorable deviation observed by the value of errors. 

### 5.2. Evaluation of GEP Models for Compressive Strength and Split Tensile Strength 

**Figure 10:**
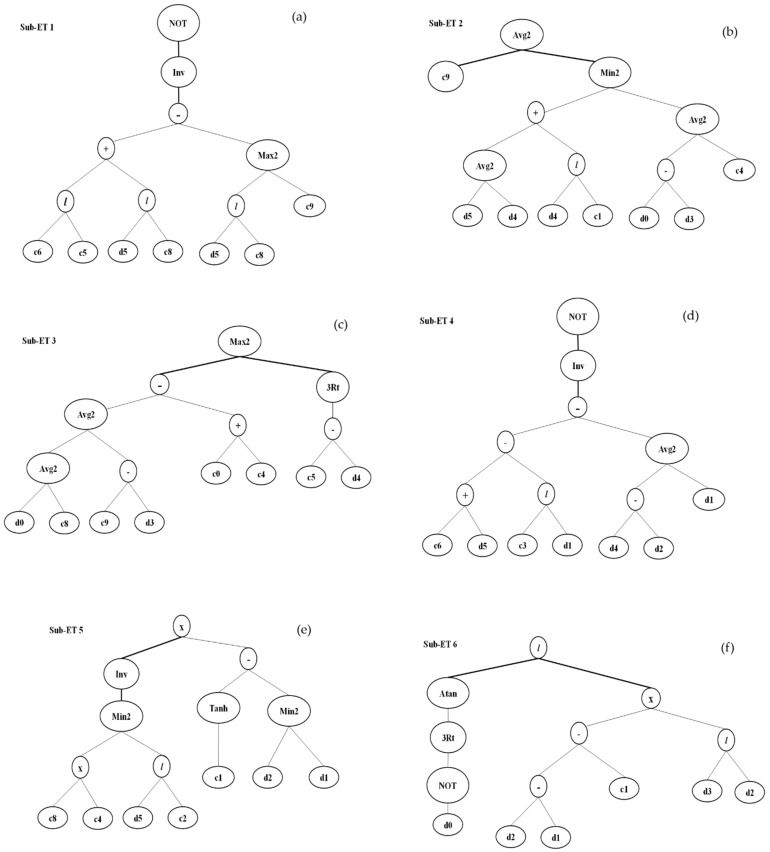
GEP expression tree for compressive strength (**a**) sub-ET 1; (**b**) sub-ET2; (**c**) sub-ET 3; (**d**) sub-ET4; (**e**) sub-ET5; (**f**) sub-ET6.

**Figure 11:**
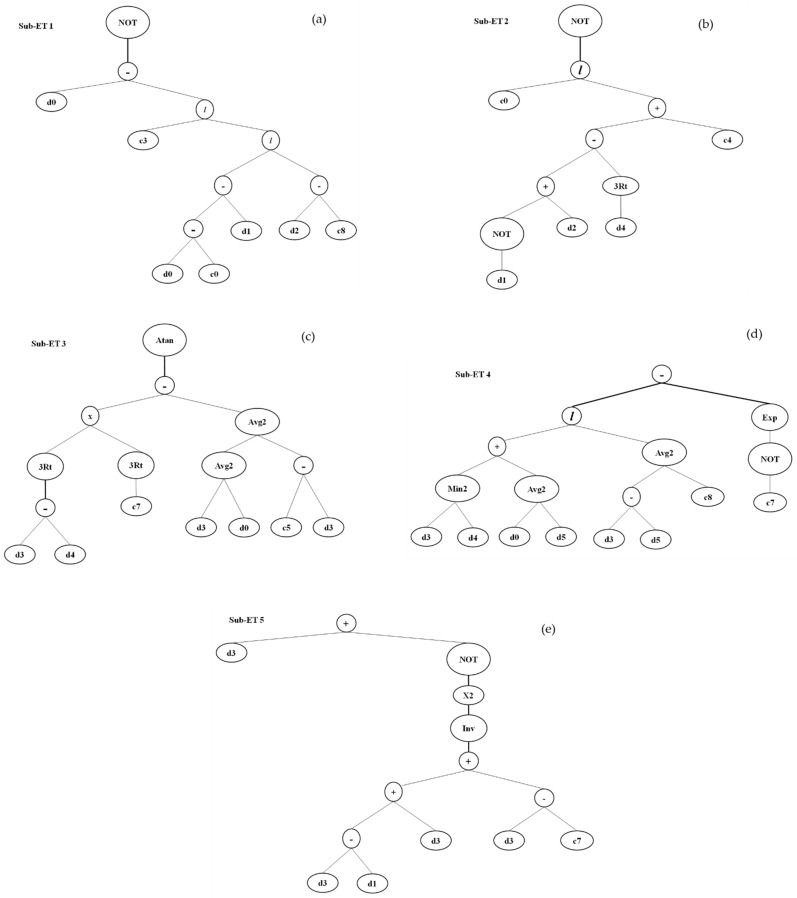
GEP expression tree for splitting tensile strength (**a**) sub-ET 1; (**b**) sub-ET2; (**c**) sub-ET 3; (**d**) sub-ET4; (**e**) sub-ET5.

The prediction and accuracy of compressive strength and split tensile strength is determined by R^2^. In this section, genetic expression models are made to predict the compressive strength and split tensile strength of concrete containing a different amount of SF. The outcome of both properties was obtained based on expression trees as shown in Figure 10 and Figure 11.

Moreover, the outcome of both properties can also be represented in the form of Equations (6) and (7), which are the empirical equations derived from ETs for each output of SFC. The ETs consist of five arithmetic operators, including. +,-,/,×,∛.

(6) $$fc\prime \left(\mathrm{MPa}\right)=A+\frac{B}{2}+C+D+E+F$$

(6a) $$A=1-\left(\frac{1}{0.485+\frac{\mathrm{CA}}{\mathrm{FA}}-2.410}\right)$$

(6b) $$B=-11.710+\frac{\mathrm{SP}+\mathrm{SF}}{2}+\frac{\mathrm{SF}}{3.954}$$

(6c) $$C=\sqrt[{3}]{\left|-3.337-\mathrm{SF}\right|}$$

(6d) $$D=1-\left(\frac{1}{-5.592+\mathrm{SP}+\frac{12.586}{\mathrm{FA}}-\frac{\mathrm{SF}-\mathrm{CA}+\mathrm{FA}}{2}}\right)$$

(6e) $$E=-\frac{1}{23.111}\;\times \;\left(-0.999-\min\left(\mathrm{CA},\mathrm{FA}\right)\right)$$

(6f) $$F=\frac{\mathrm{atan}\left(\sqrt[{3}]{\left|1-c\right|}\right)}{\left(\mathrm{CA}-\mathrm{FA}-0.6\right)\;\times \;\left(\frac{W}{\mathrm{CA}}\right)}$$

(7) $$\mathrm{fsts}\;\left(\mathrm{MPa}\right)=A+B+C+D+E$$

(7a) $$A=1-c+\frac{0.0456}{\frac{2\;\times \;c-\mathrm{FA}}{\mathrm{CA}-32.0932}}$$

(7b) $$B=1+\frac{2.8916}{1-\mathrm{FA}+\mathrm{CA}-\sqrt[{3}]{W}+4.5035}$$

(7c) $$C=\mathrm{ATan}\left(\sqrt[{3}]{\mathrm{CA}-W}\;\times \;\sqrt[{3}]{9.5013}-\left(\frac{\left(\frac{W+c}{2}\right)+7.434-W}{2}\right)\right)$$

(7d) $$D=\frac{\mathrm{SF}+\frac{c+\mathrm{SP}}{2}}{\frac{\left(W-\mathrm{SP}-37.0359\right)\;}{2}-e^{-0.276}}$$

(7e) $$E=c\;\times \;{\left(1-(\frac{1}{3W-\mathrm{FA}-10.255})\right)}^{2}$$

#### Model Outcomes of Gene Expression Programming (GEP)

**Figure 12:**
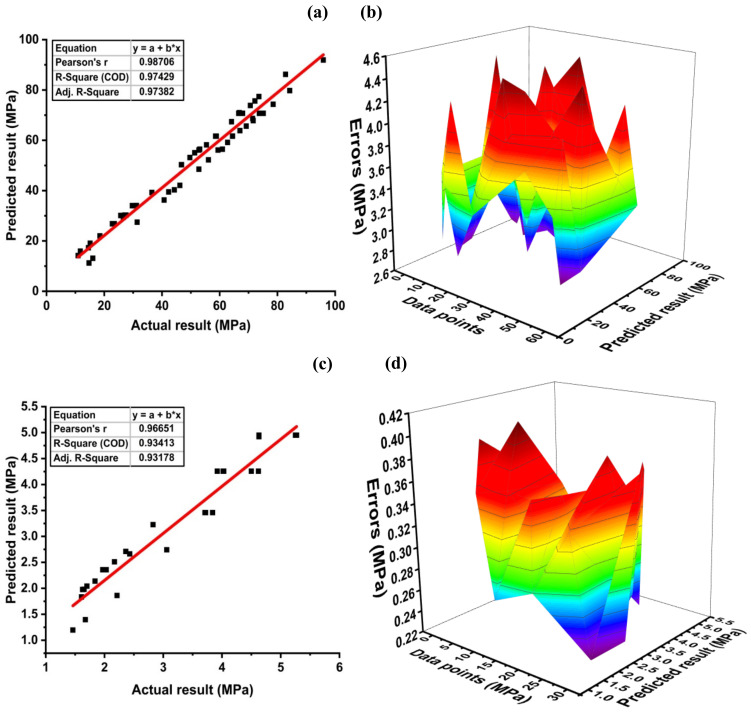
GEP model for (**a**) compressive strength and (**b**) its error distribution; (**c**) split tensile strength and (**d**) its error distribution.

GEP model performance yielded a robust relationship between actual and predicted compressive strength, as depicted in Figure 12a,b. It can be observed that R^2^, by employing GEP, is close to 1. Moreover, Figure 12b represents an average of 3.52 MPa, with the maximum and minimum errors of 4.46 and 2.70 MPa, respectively. The data of the GEP model for compressive strength indicate that all the errors lie below 5 MPa. A comparison is drawn between the actual and the predicted compressive strength to evaluate the model accuracy and to measure the deviation of the model from the experimental results. Figure 12c shows the coefficient of correlation for the split tensile strength model with R^2^ = 0.93, whereas Figure 12d shows the error distribution of predicted results with actual results. The GEP model for fst may show high error peaks as compared to the models obtained by DT, MLPNN, SVM, but the overall performance of the model is better comparatively as can be seen in the statistical parameters in Table 8. Average, maximum, and minimum errors for the tensile strength GEP model are 0.3, 0.4, and 0.23 MPa, respectively. Moreover, the data indicate that 100% of the error lies below 0.5 MPa. The expression tree for the GEP split tensile strength model is shown in Figure 11. The relationship that developed between tensile strength and input parameters is shown in Equation (7). 

### 5.3. Comparison between Ensemble Models and the GEP Model

To the author’s knowledge, no model for predicting the mechanical properties of SFC has been devised. As a consequence of this study, nonlinear regression models were developed to predict the mechanical properties of SFC, and their results were compared to gene expression models. The statistical errors between anticipated and actual values are shown in Table 8. The statistical metrics demonstrate that the actual and anticipated values are closer for GEP models, confirming their predictive ability in forecasting the compressive and split tensile strengths of SFC.

**Figure 13:**
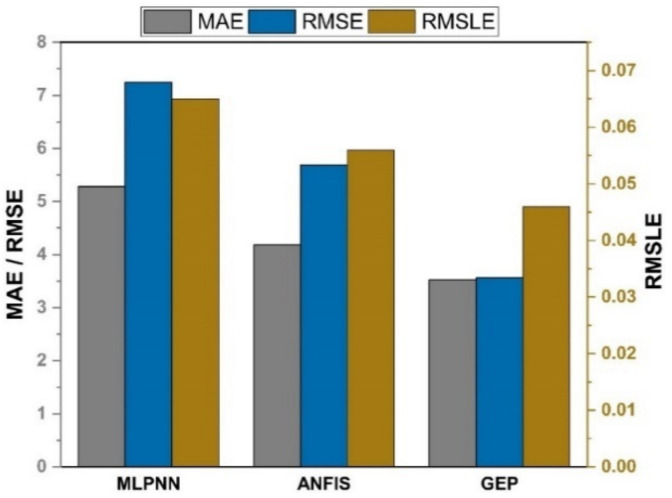
Comparison of errors for compressive strength.

**Figure 14:**
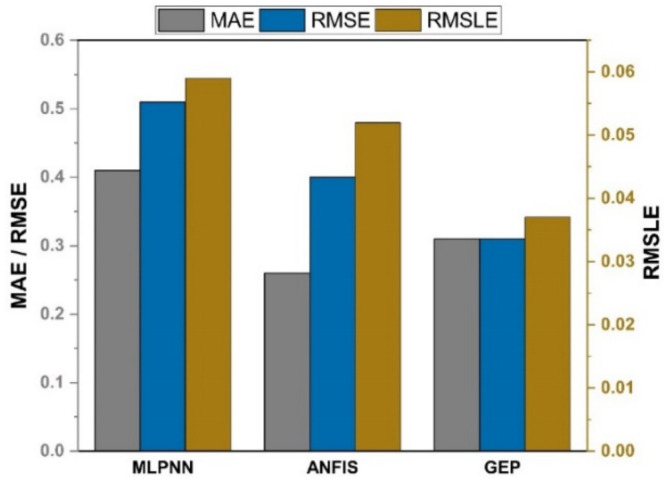
Comparison of errors for splitting tensile strength.

As shown in Figure 13 and Figure 14, the GEP models outperform other machine learning models with the same input variables for both compressive and split tensile strengths of SFC. The GEP model is superior to other machine learning models in that it is capable of effectively establishing a relationship between nonlinear input and output variables.

### 5.4. Sensitivity Analysis

**Figure 15:**
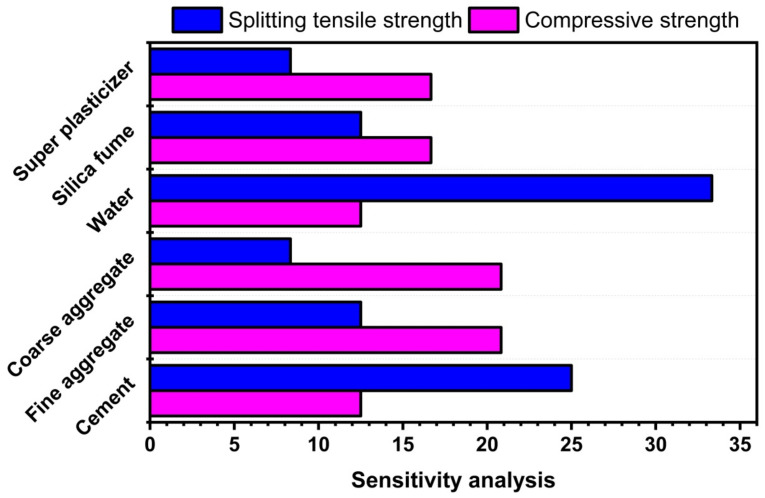
Contribution of input parameters to compressive and splitting tensile strength.

Six parameters were employed as inputs: cement, FA, CA, water, SF, and SP. The contribution of each input parameter to the construction of GEP models is depicted in Figure 15. Compressive strength is increased more by water and cement than by FA, CA, and other additives. Although, the least sensitive parameters in the creation of the tensile strength model are water and cement. The most sensitive factors for splitting tensile strength are FA and CA. Both SF and SP contributed modestly to the development of both models. 

### 5.5. Cross-Validation

Cross-validation is a statistical technique for estimating the true performance of machine learning models. It is vital to understanding the performance of the models chosen. A validation technique is required to ascertain the accuracy level of the model’s data for this purpose. The k-fold validation test requires randomly shuffling the data set and segmenting it into k-groups. The data from the experimental samples are evenly divided into ten subgroups in the given study. It makes use of nine of ten subsets, whereas the remaining one is used to validate the model. The identical procedure is then performed ten times in order to acquire the average accuracy of these ten repeats. It is commonly accepted that the tenfold cross-validation approach accurately portrays the model’s conclusion and correctness [92,93].

**Figure 16:**
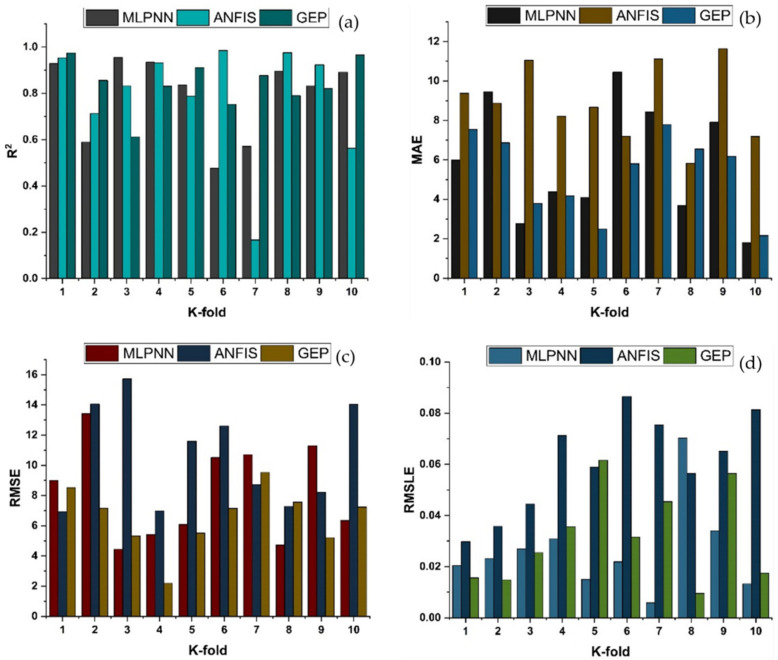
K-fold cross validation of compressive strength for MLPNN, ANFIS and GEP; (**a**) Based on R^2^; (**b**) Based on MAE; (**c**) based on RMSE; (**d**) based on RMSLE.

**Figure 17:**
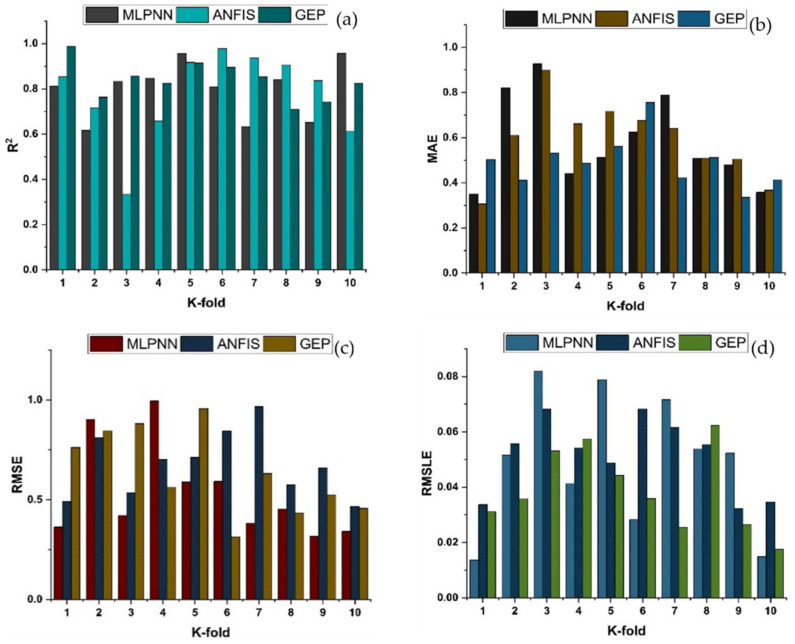
K-fold cross validation of tensile strength for MLPNN, ANFIS and GEP; (**a**) Based on R^2^; (**b**) Based on MAE; (**c**) based on RMSE; (**d**) based on RMSLE.

K-fold cross-validation can be used to check for bias and variance reduction in the test set. Correlation coefficients (R^2^), mean absolute error (MAE), mean square logarithmic error (RMSLE), and root mean square error (RMSE) are used to evaluate the outcomes of cross-validation, as depicted in Figure 16 and Figure 17 for compressive strength and splitting tensile strength, respectively. The GEP model shows fewer errors and better R^2^ as compared to supervised machine learning techniques. The average R^2^ for GEP modeling is 0.84 for a compressive strength of ten folds with maximum and minimum values of 0.97 and 0.61, as shown in Figure 16. Similarly, the average R^2^ = 0.83 for tensile strength with a maximum and minimum value of 0.98 and 0.71, respectively, is shown in Figure 17. Each model shows fewer errors for validation. The validation indicator result shows that mean values of MAE, RMSE, and RMSLE come to be 5.33, 6.54, and 0.039, respectively, for the compressive strength GEP model and 0.49, 0.63, and 0.031 for the splitting tensile strength GEP model. Similarly, the ensemble models show the same trend by showing comparatively more errors. 

## 6. Conclusions

Since the last two decades, soft computing approaches have been widely employed to forecast various properties of concrete using both linear and nonlinear modelling systems. This study used MLPNN, ANFIS, and GEP to predict the compressive and split tensile strengths of SFC. Concrete’s primary feature is compressive strength, and no model has been created to estimate the compressive strength of SFC. Following a thorough study of the literature, a substantial and reliable database was compiled from the various studies. Models were evaluated using statistical measures such as R^2^, MAE, RMSE, and RMSLE. The values of statistical parameters indicated that all models are capable of accurately predicting the compressive and split tensile strengths of concrete. The outcomes of the machine learning models and the GEP model are compared. External validation and sensitivity assessments were also done for additional assurance. R^2^ values of 0.97 for compressive strength and 0.93 for tensile strength were achieved using the best model (GEP).

The specific outcomes obtained from this study are:

1. The results of this study indicated that GEP models have higher accuracy for the prediction of data than other ML models.
2. After a detailed study, it was observed that the order of accuracy followed by the compressive strength and tensile strength models is: GEP > ANFIS > MLPNN.
3. The benefit of GEP is it gives us a new mathematical equation that can be used to predict the properties for another database.
4. Sensitivity analysis showed that water and cement are the governing factors in the model development for compressive strength. However, these factors have least effect in tensile strength model development.
5. Statistical parameters including R^2^, MAE, RMSE, and RMSLE were used to check the k-fold validation results. These parameters depicted satisfactory results for all the models.
6. Accurate expressions and models can be used to increase the industrial-level utilization of hazardous SF in concrete in construction procedures, rather than accumulating it as industrial waste. This research contributes to sustainable development by lowering energy usage, landfill waste, and greenhouse gas emissions.

## 7. Limitations and Directions for Future Work

Compressive strength and split tensile strength were determined using a comprehensive and dependable database. However, if a more generic expression is sought, adding additional input parameters and expanding the database may yield the desired results. Models developed in this study are for the prediction of SFC compressive and split tensile strength. These models provided accurate and reliable results in predicting the SFC strengths as indicated by statistical parameters. However, MLPNN, ANFIS and GEP models can be used for the prediction of concrete properties comprising various other concrete constituents by keeping the same modelling parameters. These models will be modified on the basis of input parameters and results forecasted rely mainly on the database utilized. Additionally, machine learning approaches can be used in conjunction with heuristic methods such as the whale optimization algorithm, ant colony optimization, and particle swarm optimization to achieve optimal outcomes. These strategies can then be compared to the ones used in this study.

Additionally, multi-expression programming (MEP) is a more advanced and improved variant of GEP. MEP analysis should be used to circumvent GEP’s restrictions. Comparatively, MEP employs basic decoding processes and is given special consideration when the complexity of the targeted expression is unknown. MEP can deal with exceptions, incorrect expressions, and division by zero. The gene is in charge of creating an exception, after which it changes to an arbitrary terminal symbol, resulting in no infertile learner entering the following generation. Moreover, MLPNN and ANFIS were employed in this research for the prediction of desired outcomes and employed single learners in anticipating the results. The authors recommend using ensemble ML methods where various sub-models are developed, and the best sub-model is selected using statistical parameters. 

## Figures and Tables

**Table 1:** Prediction of Concrete Properties by using Waste Material.

S. No	Algorithm Name	Notation	Dataset	Prediction Properties	Year	Waste Material Used	References
1	Artificial neural network	ANN	300	Compressive strength	2009	FA	[54]
2	Artificial neural network	ANN	80	Compressive strength	2011	FA	[55]
3	Artificial neural network	ANN	169	Compressive strength	2016	THE	[56]
4	Artificial neural network	ANN	69	Compressive strength	2017	FA	[33]
5	Artificial neural network	ANN	114	Compressive strength	2017	FA	[57]
6	An adaptive neuro-fuzzy inference system	ANFIS	55	Compressive strength	2018		[58]
7	Random Kitchen Sink algorithm	RKSA	40	V-funnel test J-ring test Slump test Compressive strength	2018	FA	[59]
8	Multivariate adaptive regression spline	M5 MARS	114	Compressive strength Slump test L-box test V-funnel test	2018	FA	[60]
9	Artificial neural network	ANN	205	Compressive strength	2019	FA GGBFS SF RHA	[61]
10	Random forest	RF	131	Compressive strength	2019	FA GGBFS SF	[62]
11	Intelligent rule-based enhanced multiclass support vector machine and fuzzy rules	IREMSVM-FR with RSM	114	Compressive strength	2019	FA	[63]
12	Support vector machine	SVM		Compressive strength	2020	FA	[64]
13	Multivariate	MV	21	Compressive strength	2020	Crumb rubber with SF	[65]
14	Biogeographical-based programming	BBP	413	Elastic modulus		SF FA SLAG	[66]
15	Support vector machine	SVM	115	Slump test L-box test V-funnel test Compressive strength	2020	FA	[67]
16	Adaptive neuro fuzzy inference system	ANFIS with ANN	7	Compressive strength	2020	POFA	[68]
17	Data envelopment analysis	DEA	114	Compressive strength Slump test L-box test V-funnel test	2021	FA	[69]

**Table 2:** Statistical Description of Data in the Model for Compressive Strength (Kg/m^3^).

Parameters	Cement	Fine Aggregate	Coarse Aggregate	Water	Silica Fume	Superplasticizer
**Statistical Description**						
Mean	393.48	702.90	1062.41	185.15	38.25	2.56
Std error	3.92	13.44	10.88	1.84	2.27	0.35
Median	383.15	653.00	1040.00	175.00	26.25	0.00
variance	4359.48	51,138.84	33,530.89	963.29	1469.97	34.80
Std. dev	66.02	226.13	183.11	31.03	38.34	5.89
Kurtosis	−0.15	−0.51	0.20	3.66	0.57	30.00
Skewness	0.15	0.11	0.61	1.50	1.11	4.97
Range	376.00	985.36	728.00	178.87	150.00	43.00
Min	224.00	184.63	702.00	135.00	0.00	0.00
Max	600.00	1170.00	1430.00	313.87	150.00	43.00
Sum	111,354.90	198,941.50	300,663.20	52,397.59	10,827.33	726.11
Count	283.00	283.00	283.00	283.00	283.00	283.00
**Training Dataset**						
Mean	393.14	697.76	1067.67	185.80	36.78	2.65
Std error	4.41	14.67	11.94	2.15	2.56	0.42
Median	382.82	653.00	1040.00	176.00	26.25	0.00
variance	4404.11	48,659.21	32,197.86	1045.27	1483.09	40.60
Std. dev	66.36	220.59	179.44	32.33	38.51	6.37
Kurtosis	−0.14	−0.38	0.28	3.70	0.52	27.05
Skewness	0.13	0.11	0.65	1.57	1.11	4.83
Range	376.00	985.37	728.00	178.88	150.00	43.00
Min	224.00	184.63	702.00	135.00	0.00	0.00
Max	600.00	1170.00	1430.00	313.88	150.00	43.00
Sum	88,848.53	157,693.90	240,1294.40	41,990.32	8313.19	599.42
Count	226.00	226.00	226.00	226.00	226.00	226.00
**Testing Dataset**						
Mean	394.85	723.64	1041.56	182.58	44.11	2.22
Std error	8.64	32.84	26.13	3.36	4.96	0.46
Median	390.00	653.00	990.00	175.00	29.62	0.00
variance	4255.64	61,470.40	38,931.08	642.77	1399.90	11.97
Std. dev	65.24	247.93	197.31	25.35	37.42	3.46
Kurtosis	−0.17	−0.93	0.05	0.46	1.07	9.21
Skewness	0.28	0.09	0.57	0.73	1.24	2.55
Range	302.00	932.82	728.00	125.70	150.00	19.00
Min	238.00	237.19	702.00	135.20	0.00	0.00
Max	540.00	1170.00	1430.00	260.90	150.00	19.00
Sum	22,506.35	41,247.52	59,368.84	10,407.28	2514.14	126.69
Count	57.00	57.00	57.00	57.00	57.00	57.00

**Table 3:** Statistical Description of Data in the Model for Split Tensile Strength (Kg/m^3^).

Parameters	Cement	Fine Aggregate	Coarse Aggregate	Water	Silica Fume	Superplasticizer
**Statistical Description**						
Mean	386.48	756.67	1102.91	186.36	55.33	3.57
Std error	4.64	23.17	18.24	3.05	12.57	0.18
Median	375.00	912.00	980.00	169.53	26.25	3.90
variance	3212.04	79,963.57	49,589.84	1388.00	23,545.85	4.98
Std. dev	56.67	282.78	222.69	37.26	153.45	2.23
Kurtosis	−0.12	−1.07	−0.83	2.72	30.06	1.27
Skewness	0.73	−0.39	0.28	1.68	5.49	0.40
Range	234.60	985.37	728.00	178.68	953.98	10.48
Min	289.49	184.63	702.00	135.20	0.00	0.00
Max	524.09	1170.00	1430.00	313.88	953.98	10.48
Sum	57,586.23	112,744.40	164,334.20	27,766.94	8244.31	531.91
Count	149.00	149.00	149.00	149.00	149.00	149.00
**Training Dataset**						
Mean	388.13	749.54	1109.50	187.36	57.08	3.55
Std error	5.79	28.83	22.72	3.85	16.18	0.22
Median	375.00	912.00	980.00	169.53	26.25	3.90
variance	3212.04	79,963.57	49,589.84	1388.00	23,545.85	4.98
Std. dev	57.92	288.26	227.22	38.52	161.78	2.22
Kurtosis	−0.32	−1.10	−0.87	2.35	27.33	1.29
Skewness	0.60	−0.33	0.18	1.61	5.25	0.38
Range	234.60	985.37	728.00	178.68	953.98	10.48
Min	289.49	184.63	702.00	135.20	0.00	0.00
Max	524.09	1170.00	1430.00	313.88	953.98	10.48
Sum	38,813.39	74,954.21	110,950.07	18,736.21	5707.68	354.82
Count	100.00	100.00	100.00	100.00	100.00	100.00
**Testing Dataset**						
Mean	383.12	771.23	1089.47	184.30	51.77	3.61
Std error	7.78	39.08	30.69	4.97	19.48	0.32
Median	375.00	920.00	980.00	165.00	26.25	3.90
variance	2966.32	74,850.46	46,145.30	1212.64	18,596.89	5.17
Std. dev	54.46	273.59	214.81	34.82	136.37	2.27
Kurtosis	0.63	−0.97	−0.61	4.14	42.05	1.47
Skewness	1.08	−0.54	0.51	1.89	6.29	0.46
Range	201.59	985.37	728.00	178.68	953.98	10.48
Min	322.50	184.63	702.00	135.20	0.00	0.00
Max	524.09	1170.00	1430.00	313.88	953.98	10.48
Sum	18,772.84	37,790.19	53,384.14	9030.73	2536.63	177.09
Count	49.00	49.00	49.00	49.00	49.00	49.00

**Table 4:** The Maximum and Minimum Range of Silica Fume Concrete Data for Compressive Strength.

Parameters	Abbreviation	Minimum	Maximum
**Input Variables**			
Binder	C	224	600
Fine Aggregate Coarse Aggregate	FA CA	184.6 702	1170 1430
Water	W	135	313.9
Silica Fume Superplasticizer	SF SP	0 0	150 43
**Output Variable**			
Compressive Strength	fc’	5.66	95.9

**Table 5:** The Maximum and Minimum Range of Silica Fume Concrete Data for Split Tensile Strength.

Parameters	Abbreviation	Minimum	Maximum
**Input Variables**			
Binder	C	289.5	524.1
Fine Aggregate Coarse Aggregate	FA CA	184.6 702	1170 1430
Water	W	135	313.9
Silica Fume Superplasticizer	SF SP	0 0	954 10.5
**Output Variable**			
Split Tensile Strength	fst’	6.97	0.66

**Table 6:** Range of Errors for Statistical Parameters [91]

Assessment Criteria	Range	Accurate Model
MAE	[0, ∞)	The Smaller the Better
RMSE	[0, ∞)	The Smaller the Better
MSLE	[0, ∞)	The Smaller the Better
R^2^ Value	(0,1]	The Bigger the Better

**Table 7:** R^2^ Values of Models.

Output Parameter	Approach Employed	R Value
Compressive Strength	MLPNN	0.85
	ANFIS	0.91
	GEP	0.97
Split Tensile Strength	MLPNN	0.90
	ANFIS	0.92
	GEP	0.93

**Table 8:** Statistical Errors in Validation Stages for the Models.

Models	MAE	RMSE	RMSLE	R^2^ Value
MLPNN				
Compressive Strength	5.28	7.25	0.065	0.85
Split Tensile Strength	0.41	0.51	0.059	0.90
ANFIS				
Compressive Strength	4.18	5.69	0.056	0.91
Split Tensile Strength	0.26	0.40	0.052	0.92
GEP				
Compressive Strength	3.52	3.56	0.046	0.97
Split Tensile Strength	0.31	0.31	0.037	0.93

## Data Availability

The data used in this study was collected form literature and is available from corresponding author upon request.
